# Multi-omics and machine learning identify GBP2 as a key therapeutic target of Qingre Kasen granules in lupus nephritis via NF-kappaB modulation

**DOI:** 10.1080/0886022X.2025.2577844

**Published:** 2025-11-06

**Authors:** Kaojiang Zhu, Rui Qian, Zhuang Huang, Xing Hong, Qi Jiang, Benjiang Xiao, Meng Chen, Yuxin Wen, Shihao Li, Pengyu Chen, Ping Huang, Qiong Wang, Fang Huang, Jingjing Li, Lintao Han

**Affiliations:** ^a^ College of Pharmacy, Hubei University of Chinese Medicine, Wuhan, China; ^b^ Public Inspection and Testing Center of Xianning, Xianning, China; ^c^ College of Basic Medical Sciences, Hubei University of Chinese Medicine, Wuhan, China; ^d^ Hubei Shizhen Laboratory, and Key Laboratory of Ministry of Education, Wuhan, China

**Keywords:** Lupus nephritis, Qingre Kasen granules, multi-omics, bioinformatics, machine learning

## Abstract

Lupus nephritis (LN), a severe renal complication of systemic lupus erythematosus (SLE), results from immune abnormalities. Qingre Kasen granules (QS), which have chicory as the main ingredient, play a significant role in treating various inflammatory and immune system diseases. This study aimed to identify LN biomarkers and mechanisms via systems biology approach, and multi-omics integration, and to screen potential active ingredients of QS for the treatment of LN. First, bioinformatics and machine learning were used to screen five genes, namely CHI3L1, CX3CR1, GBP2, CCND1, and *PKP4*, as well as the NF-kappaB signaling pathway. An external dataset was then employed to confirm that GBP2 plays a crucial role in the pathogenesis of LN. Immune infiltration analysis revealed significant changes in the proportion of CD4^+^ T cells. Subsequently, an LN mouse model was established to evaluate QS’s therapeutic effects. Results showed that QS treatment significantly improved symptoms and alleviated renal damage in these mice. Metabolomics and Western blot analyses further demonstrated that abnormal elevations of proteins related to purine metabolism and the NF-kappaB pathway were closely associated with LN’s pathogenesis. Finally, molecular docking and MMGBSA binding energy calculations identified cichoriin as a key component. Molecular dynamics simulations further predicted a strong binding affinity between cichoriin and GBP2, along with favorable ADMET properties. In summary, GBP2 is a key druggable gene in LN. This study verifies QS’s efficacy and positions cichoriin as a novel immunomodulator acting on the GBP2/NF-kappaB axis.

## 1. Introduction

Systemic lupus erythematosus (SLE) is a complex autoimmune disease caused by the inappropriate activation of the immune response, resulting in pathological changes in various systems and organs [^1^]. With a pronounced female predilection (particularly in reproductive-aged individuals), SLE imposes substantial morbidity and impairs quality of life, underscoring the urgency for targeted therapeutic strategies [^2^,^3^]. LN is a common complication of SLE. If LN cannot be diagnosed and treated in a timely manner, 5–30% of patients may develop end-stage renal disease within 10 years [^4^]. At present, the pathogenesis of LN is not yet clear. Studies have shown that it is primarily associated with factors such as genetics, infection, and immune dysfunction (B lymphocytes, T lymphocytes, macrophages, intracellular signaling molecules, cytokines, and other factors). The main goal of the treatment of lupus nephritis (LN) is to normalize renal function, or at least prevent the progressive decline of renal function. Commonly used drugs for the treatment of LN mainly include mycophenolate mofetil (MMF), rituximab (RTX), belimumab, voclosporin, and other medications [^5^]. However, there are still some drawbacks in the current treatment of LN. For example, there is incomplete remission of the disease condition, and issues such as a high recurrence rate [^6^,^7^]. This therapeutic impasse necessitates the identification of novel molecular targets and safer bioactive compounds.

The main ingredient of Qingre Kasen granules (QS) is chicory (*Cichorium intybus* L.), a plant of the genus *Cichorium* in the family Asteraceae. It is native to the Mediterranean region, Central Asia, and North Africa, and is now widely distributed in Europe, the Americas, and Asia [^8^]. Chicory contains compounds such as flavonoids, phenols, coumarin compounds, and sesquiterpene lactones [^9^]. Many researchers have found that the extract of chicory has anti-inflammatory activity, anti-hepatocellular carcinoma effects, and anti-hepatic fibrosis effect [^10^]. In existing studies, components such as sesquiterpene esters, cichoric acid, and isochlorogenic acid in chicory have demonstrated inhibitory effects on the NF-kappaB signaling pathway [^11^,^12^].

NF-kappaB signaling pathway is involved in the activation, proliferation, and differentiation of T cells and macrophages, as well as the production of immunoglobulins, and other processes, during the immune response [^13^]. These processes play a very crucial role in the pathogenesis of LN. Once activated, the NF-kappaB signaling pathway promotes the transcription of various inflammatory factors, such as interleukin-6 (IL-6), interleukin-8 (IL-8), tumor necrosis factor-α (TNF-α), and so on [^14^]. This can cause severe damage to various organs. In view of the important role of NF-kappaB in the pathogenesis of LN, the treatment strategy targeting NF-kappaB has potential application prospects. Among the various regulatory factors of NF-kappaB, guanylate-binding protein 2 (GBP2), a key interferon-γ (IFN-γ)-inducible protein, has been confirmed to be closely associated with cell-autonomous immunity, inflammatory responses, and cell apoptosis [^15^]. Recent studies have shown that GBP2 is also involved in the pathogenesis of chronic inflammation, autoimmune diseases, and cancer, suggesting that it may possess broad-spectrum inflammatory regulatory functions [^16^,^17^]. However, the specific role of GBP2 in lymph nodes remains to be further explored.

In this study, we first obtained datasets of LN patients and healthy individuals from the GEO database, screened candidate genes using WGCNA and machine learning, followed by validation in an independent dataset. We then employed CIBERSORT to analyze the immune microenvironment in LN patients. By establishing an MRL/Lpr mouse model, we validated the effect of QS in ameliorating renal inflammation and associated protein changes in LN mice. Combined metabolomics analysis was used to observe alterations in key metabolites and metabolic pathways in LN mice, exploring the underlying mechanisms. Finally, through a comprehensive strategy involving molecular docking, MMGBSA binding energy calculation, molecular dynamics (MD) simulation, and ADMET property analysis, QS components predicted to be active for LN treatment were identified. Relevant results provide valuable data support and reference for computer-aided drug research and development.

## 2. Materials and methods

### 2.1. Statistical power analysis and sample size

To determine sample size requirements, a statistical power analysis was performed using G*Power software (version 3.1.9.2). A two-tailed paired *t*-test (means: difference between two dependent means (two groups)) was selected with the following parameters: effect size (*d*) = 0.5, significance level (*α*) = 0.05, and statistical power (1 − *β*) = 0.8. This calculation yielded a minimum required sample size of *n* = 64. Considering that this study is an exploratory preclinical mechanism research and supplemented by multiple technical validations (e.g., triple replicates for Western blot), the final sample size was set to six mice per group.

### 2.2. Experimental animals and EthicsTwelve

Thirteen-week-old female specific pathogen-free (SPF) grade MRL/Lpr mice (LN model) were purchased from Chengdu Lanbo Biotechnology Co., Ltd. (Chengdu), and six age-matched C57BL/6J mice (wild-type controls) were purchased from Wuhan Zikeheng Biotechnology Co., Ltd. (Wuhan, China). All mice were acclimatized for 1 week under standardized SPF conditions (temperature: 22 ± 2 °C, humidity: 50 ± 10%, 12 h/12 h light/dark cycle) before the experiment. This study was approved by the Experimental Animal Ethics Committee of Hubei University of Chinese Medicine (approval number: HBUCMS202412009) and strictly adhered to the principles of animal welfare.

### 2.3. Experimental design and grouping

This study adopted a randomized, double-blind, and controlled design. Twelve MRL/Lpr mice were randomly divided into two groups (*n* = 6 per group) using a computer-generated random number sequence (generated using R package ‘randomizeR’ v2.1.0 (R Foundation for Statistical Computing, Vienna, Austria) with a seed number set to 12345): the model group and the QS treatment group. Additionally, six C57BL/6J mice were set as the normal control group. The group allocation was performed by independent researchers who were not involved in subsequent experimental operations to ensure allocation concealment.

### 2.4. Administration protocol and intervention

Experimental intervention was conducted when the mice were 14–16 weeks old, lasting for 21 days. QS (Xinjiang Uygur Pharmaceutical Co., Ltd. (Ürümqi, China), National Medical Products Administration Approval No. Z65020172) was dissolved in 0.2 mL of sterile normal saline at a dose of 0.755 g/kg/day, and administered intragastrically to mice in the QS treatment group daily. Mice in the model group and normal control group received 0.2 mL of sterile normal saline intragastrically each day.

### 2.5. Applicability of reporting guidelines

This study strictly adheres to the animal experiment reporting standards specified in the ARRIVE 2.0 Guidelines and employs the TRIPOD-ML checklist to achieve transparent reporting of machine learning feature selection [^18^,^19^]. The complete ARRIVE 2.0 and TRIPOD-ML checklists are presented in Supplementary Tables S1 and S2, respectively.

### 2.6. Data collection and download

**Figure 1.**
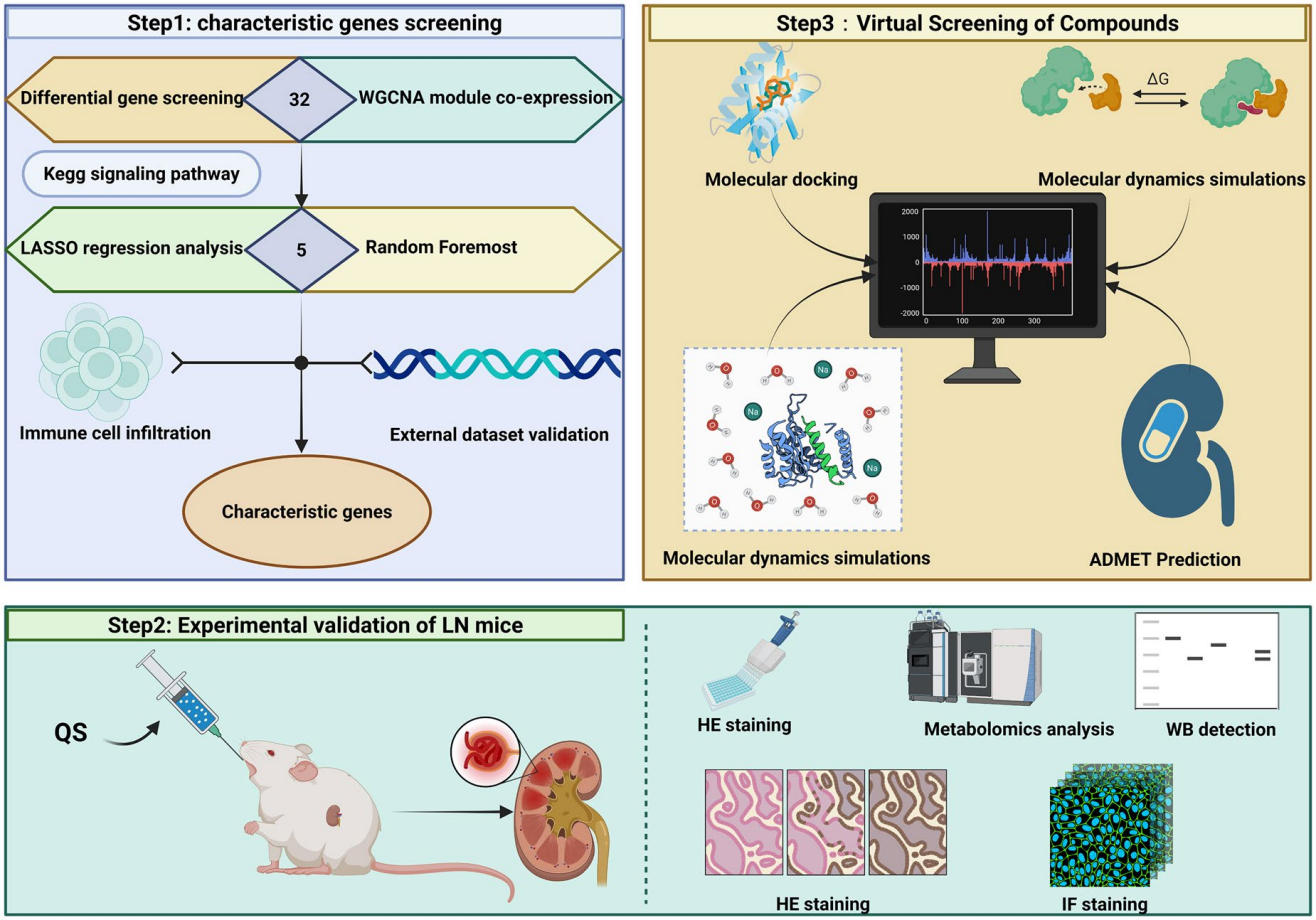
Research roadmap.

The gene expression data of LN were collected from the Gene Expression Omnibus (GEO) database (https://www.ncbi.nlm.nih.gov/geo/). The GSE32591 dataset file (a total of 46 samples, including 14 control samples and 32 LN samples) was downloaded from the GPL14663 platform, and the GSE224705 dataset (a total of 448 samples, including 20 control samples and 428 LN samples) was downloaded from the GPL13158 platform. The research roadmap is shown in Figure 1.

### 2.7. Data preprocessing and screening of differentially expressed genes (DEGs)

DEGs between the LN disease group and control group were screened using the criteria of adjusted *p* < 0.05 and |log_2_(fold change, FC)| > 1. The top 10 upregulated and 10 downregulated DEGs with the most significant FCs were selected to construct a hierarchical clustering heatmap visualizing their expression profiles.

### 2.8. Functional enrichment analysis

All expressed genes underwent Kyoto Encyclopedia of Genes and Genomes (KEGG) pathway enrichment analysis using the DAVID database (https://davidbioinformatics.nih.gov) to look for the biological functions and signaling pathways associated with LN-related DEGs.

### 2.9. Weighted gene co-expression network analysis

Weighted gene co-expression network analysis (WGCNA) was conducted using the WGCNA package in R software. First, the gene expression matrix from the GSE32591 dataset was preprocessed by filtering out low-expressed genes (defined as having FPKM values < 1 in ≥90% of samples), and converted to the topological overlap matrix (TOM). During module identification, a dynamic tree-cutting algorithm was applied with parameters set as follows: minimum module size of 50 genes, deep split height threshold of 0.99, and a merging threshold for highly correlated modules (based on topological overlap similarity) of 0.25. Finally, module eigengenes were used to characterize the expression patterns at the module level, and module membership (MM) values were calculated to quantify the correlation between individual genes and their respective modules.

### 2.10. LASSO and RandomForest were used to screen for characteristic genes

The glmnet package in R was employed to perform L1 regularization on standardized gene expression data. The optimal penalty parameter (*λ*) was selected using 10-fold cross-validation, with the criterion of minimizing the standard error (*λ*_1_se). Genes with absolute regression coefficients (|*β*|) > 0.01 were retained as characteristic genes for subsequent analysis. The RandomForest package was used to construct a random forest model, where the optimal number of features was determined by evaluating the average out-of-bag error rate across candidate genes. Genes ranking in the top 5% of importance scores, as measured by the MeanDecreaseAccuracy metric, were shortlisted. The intersection of these top-ranked genes with the LASSO-derived characteristic genes was then extracted to identify core hub genes critical for model performance.

### 2.11. Validation of feature genes, GSEA analysis, and PPI network construction

Expression validation of candidate signature genes was performed using the external dataset GSE224705. Subsequently, gene set enrichment analysis (GSEA) was conducted on the NF-kappaB signaling pathway gene set within the GSE32591 dataset to identify consistent expression patterns of target genes and NF-kappaB pathway-related genes in LN patients. Genes derived from the above screening (key signature genes and NF-kappaB pathway components) were used to construct a PPI network via the STRING database (https://string-db.org).

### 2.12. Immune cell infiltration assessment

Using the CIBERSORT algorithm, we deconvolved the expression matrix of immune cell subtypes based on the principle of linear support vector regression to evaluate the relative proportions of 22 types of immune cells in the samples of LN patients and the control group. Specifically, we utilized the CIBERSORT package in R software, combined with the LM22 gene set, to infer the infiltration status of various immune cells in the samples. We then demonstrated the differences in immune cell infiltration between LN patients and healthy individuals and plotted box-plots.

### 2.13. Serum ELISA analysis

Mice were anesthetized with isoflurane (2–3% induction, 1–2% maintenance) to minimize stress. Blood was collected via retro-orbital puncture using sterile capillaries, transferred to centrifuge tubes, and serum was separated by centrifugation at 3,000 × *g* for 15 min at 4 °C. Serum samples were stored at −80 °C until use. Commercial ELISA kits were used to detect TNF-α, IL-1β, dsDNA antibody, and ANA according to the manufacturer’s protocols. Assays were performed on a microplate reader at the recommended wavelength.

### 2.14. Renal hematoxylin–eosin (HE) staining

Mouse kidney tissues were fixed in 4% paraformaldehyde, dehydrated sequentially with ethanol of different concentrations, and then cleared with xylene before paraffin embedding. Sections were cut and dewaxed, followed by staining with hematoxylin–eosin. Finally, observe the slices under a microscope and take photographs.

### 2.15. Immunofluorescence (IF) staining

Renal tissue sections were fixed with 4% paraformaldehyde and subjected to antigen retrieval in citrate buffer. After blocking with 5% BSA for 30 min, the sections were incubated with diluted GBP2 primary antibody at 4 °C overnight. Following PBS washes, fluorescent secondary antibody was added and incubated at 37 °C in the dark for 1 h. The nuclei were stained with DAPI, and the sections were mounted. Observation was performed using a fluorescence microscope. Finally, ImageJ software (Bethesda, MD) was used for analysis, with the threshold set to 50, to determine the total fluorescence intensity.

### 2.16. Metabolomic analysis

Fifty milligrams of kidney tissue stored at −80 °C was thawed at room temperature and minced into small pieces. Transfer the minced tissue to a centrifuge tube, add ice-cold 80% methanol, and homogenize thoroughly. Sonicate the mixture on ice, then centrifuge at 13,000 × *g* for 20 min at 4 °C. Transfer the supernatant to a new tube and dry by centrifugal concentration at 35 °C for 2 h. Resuspend the dried pellet in 40 μL of methoxyamine hydrochloride solution (40 mg/mL, dissolved in pyridine) and 10 μL of d27-myristic acid internal standard (0.75 mg/mL, prepared in pyridine). Incubate the mixture at 37 °C for 90 min, then add 50 μL of MSTFA (containing 1% TMCS), and incubate at 70 °C for 60 min. After cooling, centrifuge at 13,000 × *g* for 10 min and transfer the supernatant to a GC–MS vial for analysis. The raw data were pre-processed using AMDIS software, including peak identification and normalization, to generate matrices.

Data analysis and screening of differential metabolites are carried out by conducting partial least squares discriminant analysis (PLS-DA) through the MetaboAnalyst 6.0 website (https://dev.www.metaboanalyst.ca/). Finally, key differential metabolites and metabolic pathways were screened.

### 2.17. Determination of relevant proteins by Western blot

Kidney tissue was placed on ice, lysed with RIPA lysis buffer, and the supernatant was collected after centrifugation at 12,000 r/min for 15 min. Protein concentration was determined by the BCA method. Equal amounts of protein samples (30–50 μg) were separated by 10–12% SDS-PAGE (stacking gel 80 V, separating gel 120 V) and transferred to PVDF membranes (250 mA, 90 min) by wet transfer. After blocking the membrane with 3% BSA, incubate the primary antibody overnight at 4 °C and incubate the HRP-conjugated secondary antibody for 1 h after washing. Finally, the protein bands were detected using ECL reagents and a chemiluminescence imaging system.

### 2.18. Molecular docking and MMGBSA binding free energy calculation for screening potential active compounds

Based on the GBP2 protein screened above, obtain the detailed characteristics of its protein structure through the PDB website (https://www.rcsb.org/). In this study, information related to QS-related components has been obtained through the preliminary work of the experimental group [^20^]. Obtain SDF files for QS-related components and inhibitors from the PubChem website (https://pubchem.ncbi.nlm.nih.gov/). First, use the Proteins.Plus website (https://proteins.plus/) to predict the binding pocket of the GBP2 protein. Subsequently, import the prepared molecular structure files of the GBP2 protein and QS-related components into Schrodinger software (version 12.8), process them into the format required for docking, and then molecular docking and MMGBSA binding free energy calculation were performed, with comparisons made with inhibitors. Finally, compare with the amino acid residues corresponding to GBP2 protein family inhibitors to screen for optimal compounds, and use PyMOL software (version 4.6) for visualization.

### 2.19. Molecular dynamics simulation

To further evaluate the binding stability of the screened compounds with GBP2, MD simulations were performed using GROMACS software (version 2020.3_GPU). A 40 ns MD simulation was conducted on the docked target ligand–GBP2 protein complex, generating various simulation results from the trajectories, which were then compared with those of inhibitors.

### 2.20. ADMET property prediction

ADMET properties (absorption, distribution, metabolism, excretion, and toxicity) are critical parameters for evaluating the drug-likeness potential of candidate compounds during drug discovery and development. They directly influence the compound’s *in vivo* efficacy and safety. To systematically predict the *in vivo* processes and potential risks of cichoriin, this study employed computer simulation methods to comprehensively assess its ADMET properties. Specifically, using the ADMETlab 3.0 software platform, a thorough predictive analysis was conducted on cichoriin’s intestinal absorption capacity, *in vivo* distribution characteristics, hepatic metabolic pathways, excretion efficiency, and potential toxicity. This aimed to obtain key parameters related to its ADMET profile, providing a theoretical basis for subsequent research.

### 2.21. Statistical analysis

All statistical analyses in this study were performed using R software (version 4.4.3). Unless otherwise specified, *p* < 0.05 was considered statistically significant.

## 3. Results

### 3.1. Identification of differentially expressed genes and functional enrichment analysis

**Figure 2.**
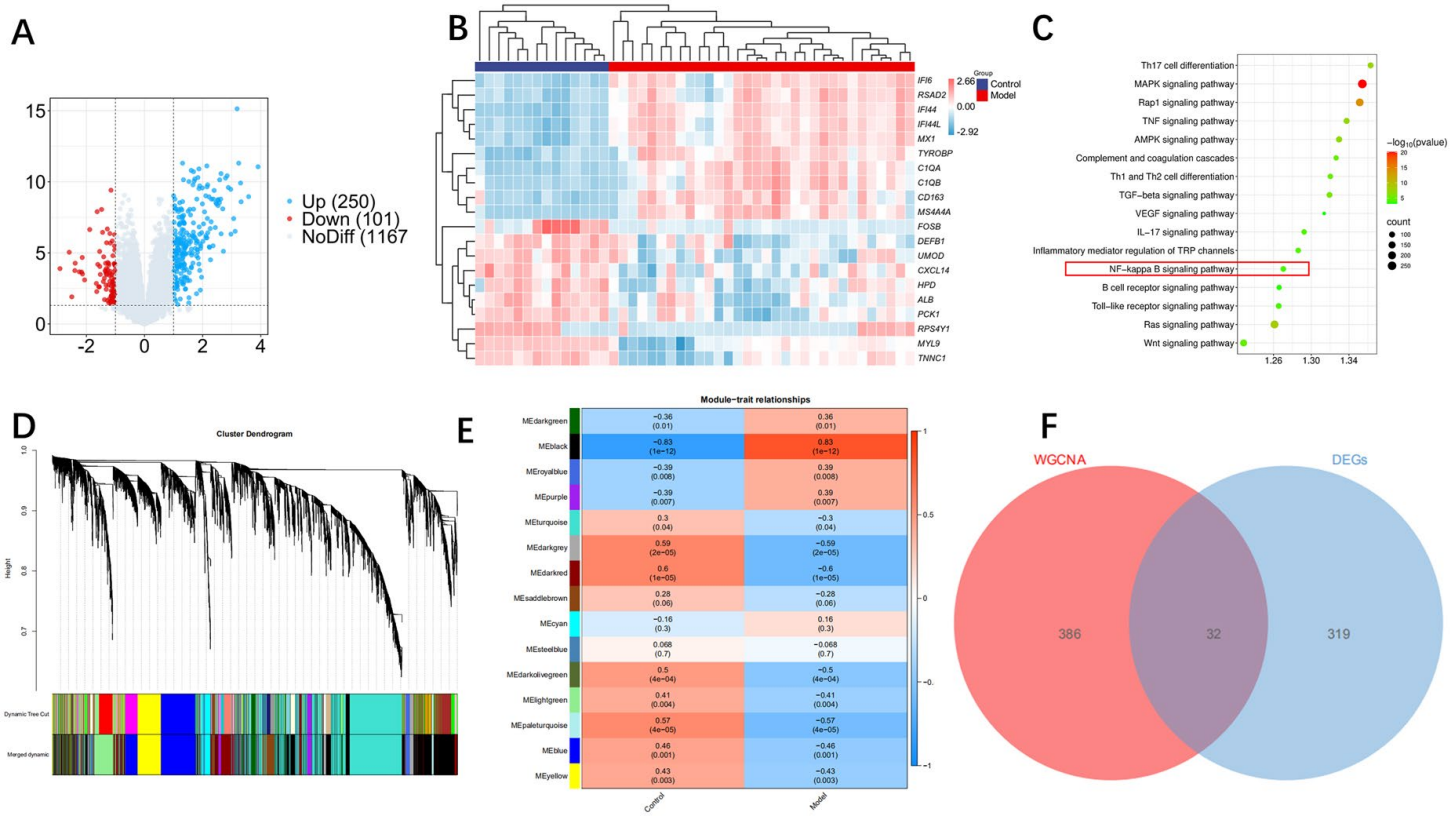
Identification of lupus nephritis-related genes and pathways. (A) Volcano plot of differentially expressed genes between patients with LN and healthy individuals. (B) Analysis of the heatmap of the associations of differentially expressed genes. (C) KEGG analysis of the expressed genes in patients with LN. (D) Gene systematic clustering tree and corresponding modules drawn based on TOM. (E) Heatmap of the correlation between modules and traits/groups. (F) Venn diagram of differentially expressed genes and genes in the key modules of WGCNA.

A total of 351 DEGs were identified from renal tissue transcriptome data by comparing the control and model groups, including 250 upregulated and 101 downregulated genes (Figure 2(A)). Select the top 10 upregulated genes and top 10 downregulated genes with the largest FCs in differential expression for visualization in a hierarchical correlation heatmap, highlighting distinct expression patterns between groups (Figure 2(B)). KEGG pathway enrichment analysis revealed significant enrichment of biological pathways closely associated with LN pathogenesis, including the NOD-like receptor signaling pathway, Toll-like receptor signaling pathway, Th17 cell differentiation pathway, PI3K–Akt signaling pathway, and NF-kappaB signaling pathway (Figure 2(C)).

### 3.2. Construction of weighted gene co-expression network

WGCNA clustered all genes into 23 co-expression modules (Figure 2(D,E)). Modules with correlation coefficients closest to extreme values (blue for negative correlation, red for positive correlation) were identified as characteristic modules distinguishing LN patients from normal controls. The MEblack module showed the strongest associations with LN, with correlation coefficient of −0.83. To prioritize key genes, a Venn diagram analysis was performed to intersect genes in WGCNA characteristic modules with DEGs, yielding 32 candidate hub genes (Figure 2(F)).

### 3.3. Selection of characteristic genes through LASSO and random forest algorithms

**Figure 3.**
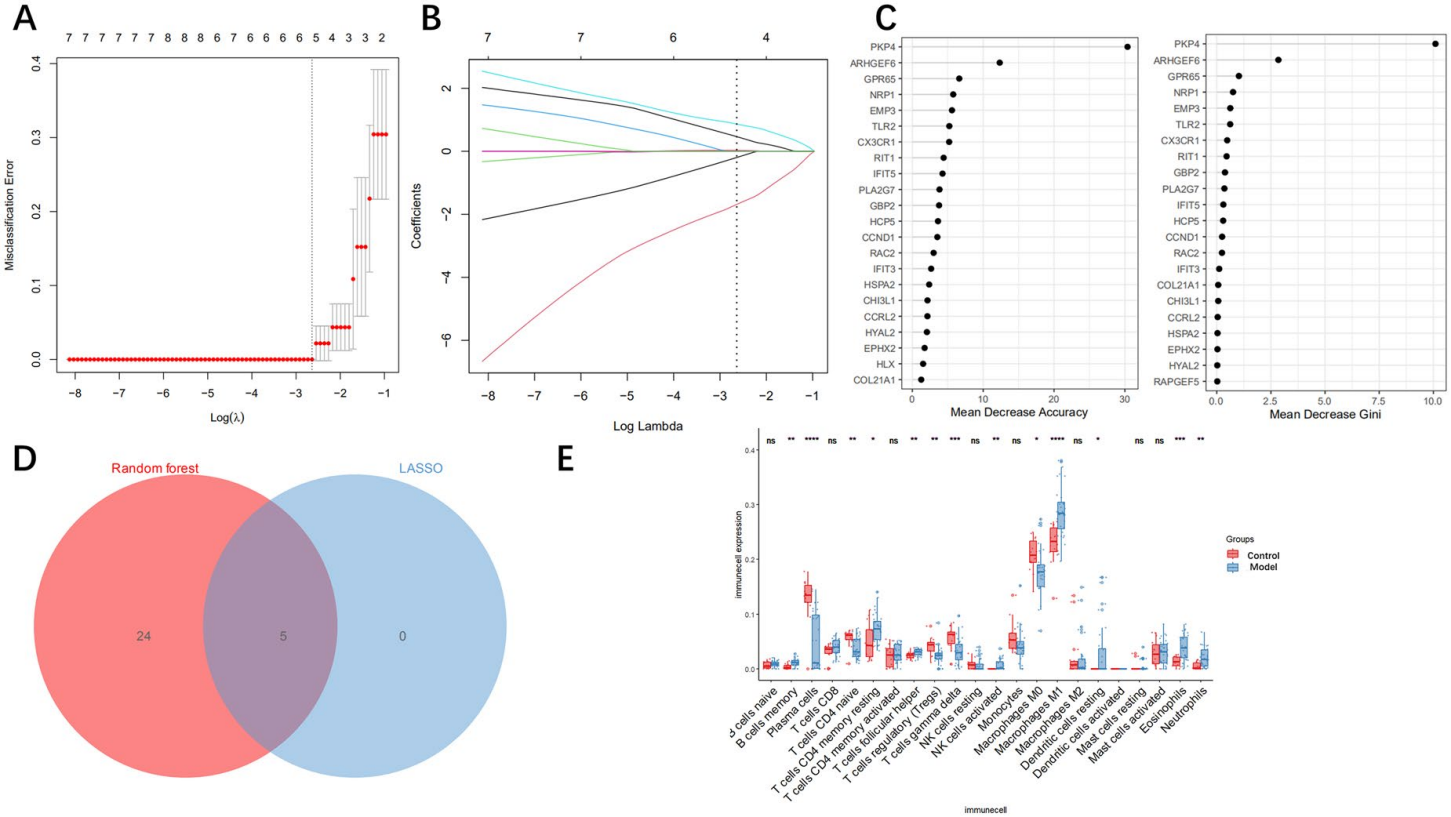
Screening of characteristic genes by machine learning. (A) Penalty plot of the LASSO model. (B) The LASSO plot shows that as the *k* penalty value increases, the magnitude of the parameter coefficients also decreases. (C) Ranking of important genes in the random forest model. (D) Intersection genes obtained by the LASSO algorithm and the random forest algorithm. (E) Immune cell infiltration in healthy individuals and LN patients. ‘ns’ indicates *p* ≥ 0.05; *p < 0.05; **p < 0.01; ***p < 0.001; ****p < 0.0001.

Two machine learning algorithms were applied to screen feature genes from MEblack module genes. For the LASSO analysis, five characteristic genes corresponding to the minimum error were selected (Figure 3(A,B)). According to the criteria of Accuracy > 1 and Gini index > 0.5, 29 characteristic genes corresponding to the random forest algorithm were selected. Finally, through the intersection of the two algorithms, CHI3L1, CX3CR1, GBP2, CCND1, and PKP4 were ultimately determined as the characteristic genes (Figure 3(D)).

### 3.4. Analysis of immune cell infiltration

In order to explore the differences of 22 subtypes of immune cells between healthy individuals and LN patients, we analyzed the data by using the method of immune cell infiltration. Compared with the control group, B cells memory, plasma cells, T cells CD4 naive, T cells follicular helper, T cells regulatory (Tregs), T cells gamma delta, NK cells activated, macrophages M1, eosinophils, and neutrophils showed significant changes in the model group (Figure 3(E)). Among them, plasma cells, Tregs, and T cells gamma delta showed a downward trend in LN patients, while B cells memory, T cells follicular helper, NK cells activated, macrophages M1, eosinophils, and neutrophils showed an upward trend.

### 3.5. Expression validation and pathway network analysis

**Figure 4.**
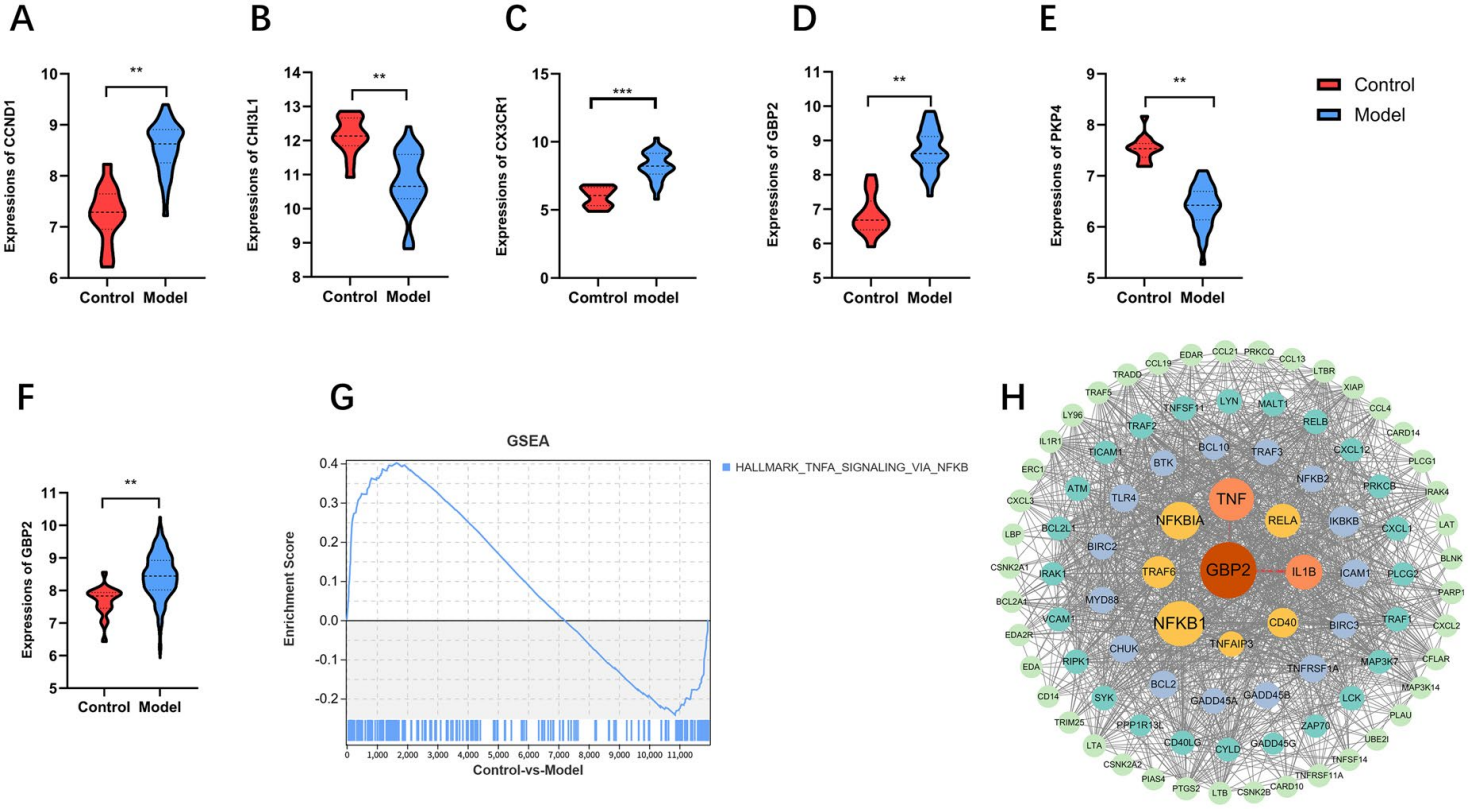
Expression and validation of characteristic genes in LN patients. (A–E) Expression trends of five characteristic genes in the training set GSE32591. (F) Expression trend of GBP2 in the validation set GSE224705. (G) NF-kappaB signaling pathway in GSEA analysis. (H) PPI network of genes in GBP2 and NF-kappaB signaling pathway. **p < 0.01; ***p < 0.001.

Expression levels of CHI3L1, CX3CR1, GBP2, CCND1, and PKP4 were analyzed in the GSE32591 dataset. CX3CR1, CCND1, and GBP2 exhibited significantly increased expression in LN patients, whereas CHI3L1 and PKP4 were downregulated (Figure 4(A–E)). Using the GSE224705 dataset for external validation, only GBP2 remained significant (Figure 4(F)). Given the established role of NF-kappaB signaling in LN pathogenesis, GSEA of the NF-kappaB pathway in the GSE32591 training set revealed significant pathway activation in LN patients (Figure 4(G)). A PPI network was constructed for GBP2 and NF-kappaB pathway components using STRING (confidence score > 0.7), identifying strong functional associations between GBP2 and the inflammatory cytokines TNF-α and IL-1β, which are key effectors of NF-kappaB signaling (Figure 4(H)).

### 3.6. Metabolomics discovery for biomarker identification

**Figure 5.**
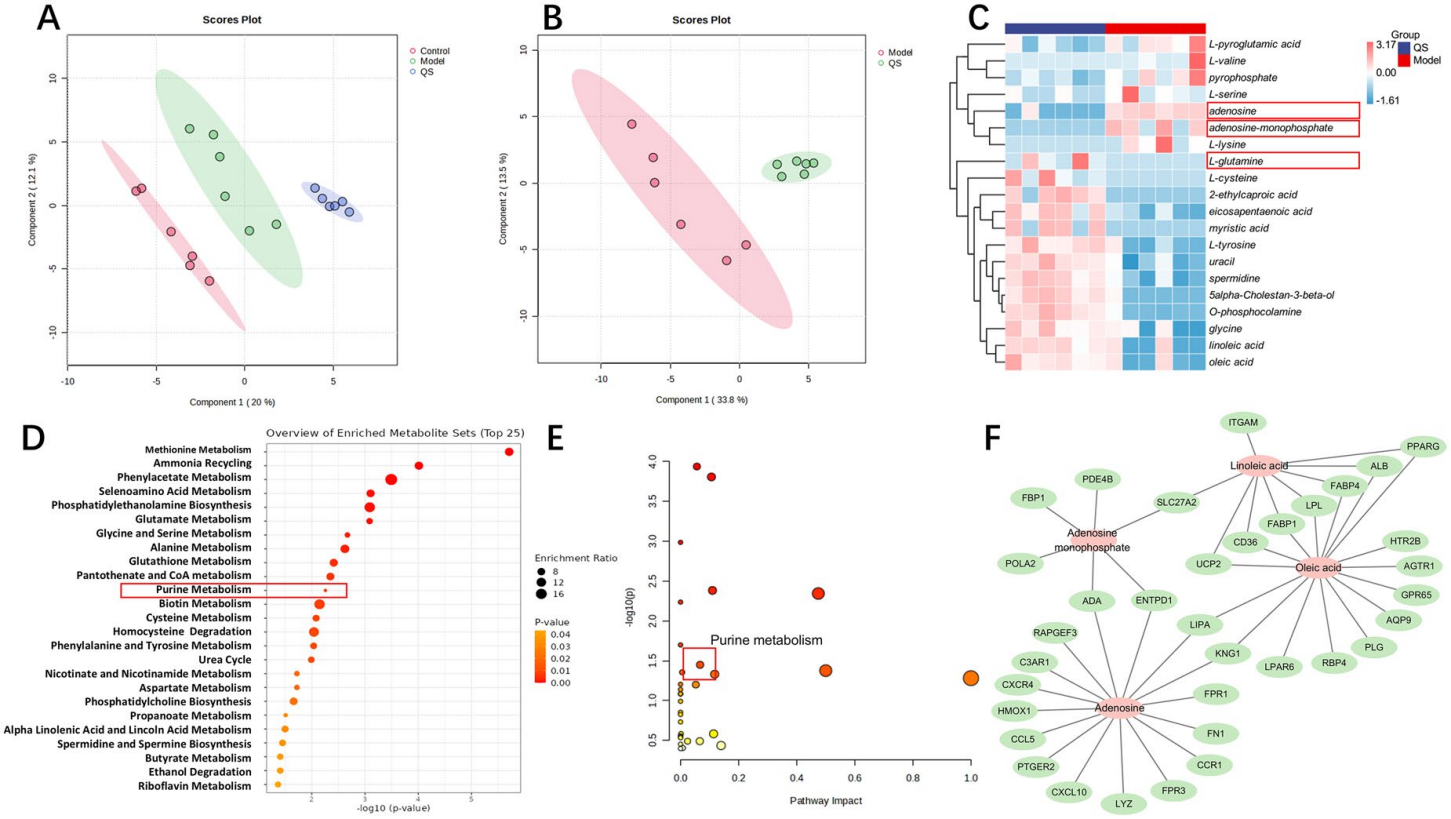
Metabolomics of QS in the treatment of LN mice. (A) PLS-DA distribution diagram of the three groups. (B) PLS-DA plots of the model group and the QS group. (C) Interaction heatmap of differential metabolites between the normal group and the model group. (D) Potential metabolic pathways in LN mice under treatment. (E) Bubble chart of the pathway analysis of differential metabolites. (F) Correlation diagram of differential genes and differential metabolites. VIP > 1.0 and *p* < 0.05.

PLS-DA was performed on the kidney tissue samples from three groups of mice. The results showed that the metabolic profiles exhibited distinct clustering, indicating that there were significant differences in the metabolite composition among the groups (Figure 5(A,B)). Comparative analysis between the model and QS groups identified 20 differential metabolites, including 13 upregulated metabolites (l-glutamine, l-cysteine, 2-ethylcaproic acid, eicosapentaenoic acid, myristic acid, l-tyrosine, uracil, spermidine, 5alpha-cholestan-3-beta-ol, O-phosphocolamine, glycine, linoleic acid, and oleic acid) and seven downregulated metabolites (l-pyroglutamic, l-valine, pyrophosphate, l-serine, adenosine, adenosine-monophosphate, and l-lysine) (Figure 5(C)). These metabolites met statistical significance criteria (variable importance in the projection (VIP) > 1, *p* < 0.05). Subsequent metabolic pathway analysis identified enrichment in pathways such as methionine metabolism, glutamate metabolism, and purine metabolism (*p* value < 0.05) (Figure 5(D,E)). A joint analysis of differential genes and metabolites was then performed. Correlation analysis showed that metabolites including adenosine, adenosine monophosphate, oleic acid, and linoleic acid were most closely associated with differential genes (Figure 5(F)).

### 3.7. Serum biomarker analysis and histopathological examination

**Figure 6.**
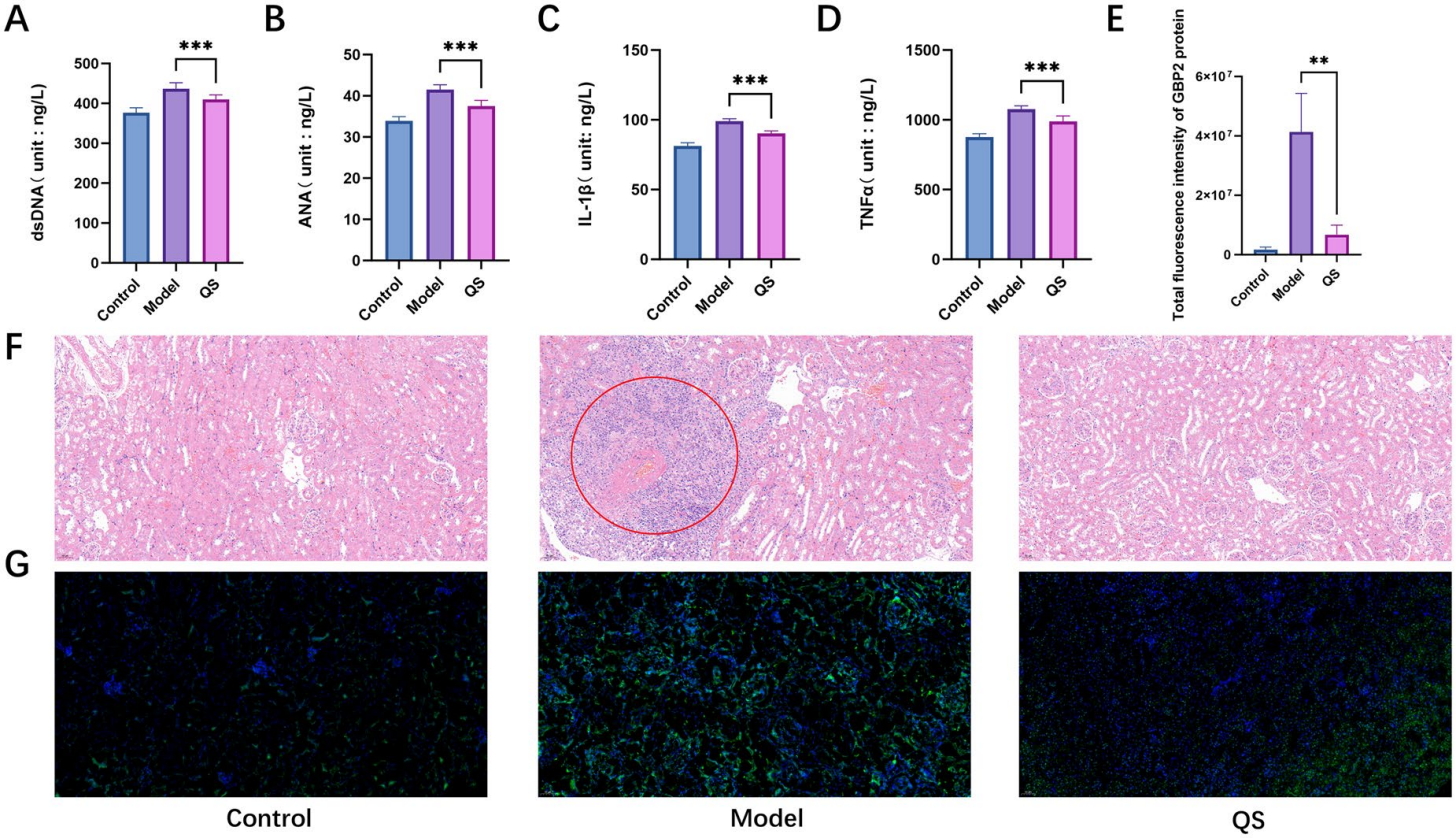
QS treatment ameliorates disease phenotypes and renal pathology in LN mice. (A, B) Detection of the levels of dsDNA antibody and ANA antibody in the serum by ELISA. (C, D) Detection of the levels of inflammatory factors in the serum by ELISA. (E) The total fluorescence intensity of GBP2 was analyzed using ImageJ software. (F) Observation of kidney tissue sections by HE staining. (G) Immunofluorescence (IF) staining showing GBP2 expression (green) in renal tissue. The scale bar is 50 μm. **p < 0.01, ***p < 0.001; *n* = 6, indicating statistically significant intergroup differences.

Compared to the model group, the QS group exhibited significantly lower serum levels of anti-double-stranded DNA antibody (anti-dsDNA) (mean difference = −26.55 pg/mL, 95% CI [−39.95, −13.15], Cohen’s *d* = 1.95, *p* < 0.001), antinuclear antibody (ANA) (mean difference = −4.01 pg/mL, 95% CI [−5.32, −2.70], Cohen’s *d* = 2.75, *p* < 0.001), and inflammatory cytokines interleukin-1β (IL-1β) (mean difference = −8.77 pg/mL, 95% CI [−10.61, −6.93], Cohen’s *d* = 4.83, *p* < 0.001) and TNF-α (mean difference = −87.62 pg/mL, 95% CI [−121.3, −53.93], Cohen’s *d* = 2.78, *p* < 0.001) (Figure 6(A–D)).

Pathological morphology observations revealed that under H&E staining, the renal tissue of mice in the control group exhibited intact glomerular structure, distinct and well-arranged cellular layers, and no edema or inflammatory cell infiltration in the renal interstitium. In contrast, the model group showed significant pathological alterations: collagen fiber proliferation and mild fibrosis were observed in the renal interstitium, accompanied by aggregates of numerous hyperplastic cells exhibiting deep blue staining. Following QS intervention, these pathological injuries were significantly alleviated, with reduced interstitial fibrosis and a decreased number of hyperplastic cells (Figure 6(E)).

The IF staining results indicated that the expression level of GBP2 protein differed among the groups: the fluorescence intensity of GBP2 in the renal tissue of mice in the model group was significantly enhanced, suggesting a marked increase in its total amount; after QS intervention, the fluorescence intensity of GBP2 was significantly weaker compared with the model group (mean difference = −36,713,855.33, 95% CI [−11,646,788.9, −61,780,921.7], Cohen’s *d* = 3.33, *p* < 0.05), indicating that its total amount was effectively reduced (Figure 6(E,G)).

### 3.8. QS inhibited the expression of IFN-γ/JAK1/STAT1/GBP2/NF-kappaB protein

**Figure 7.**
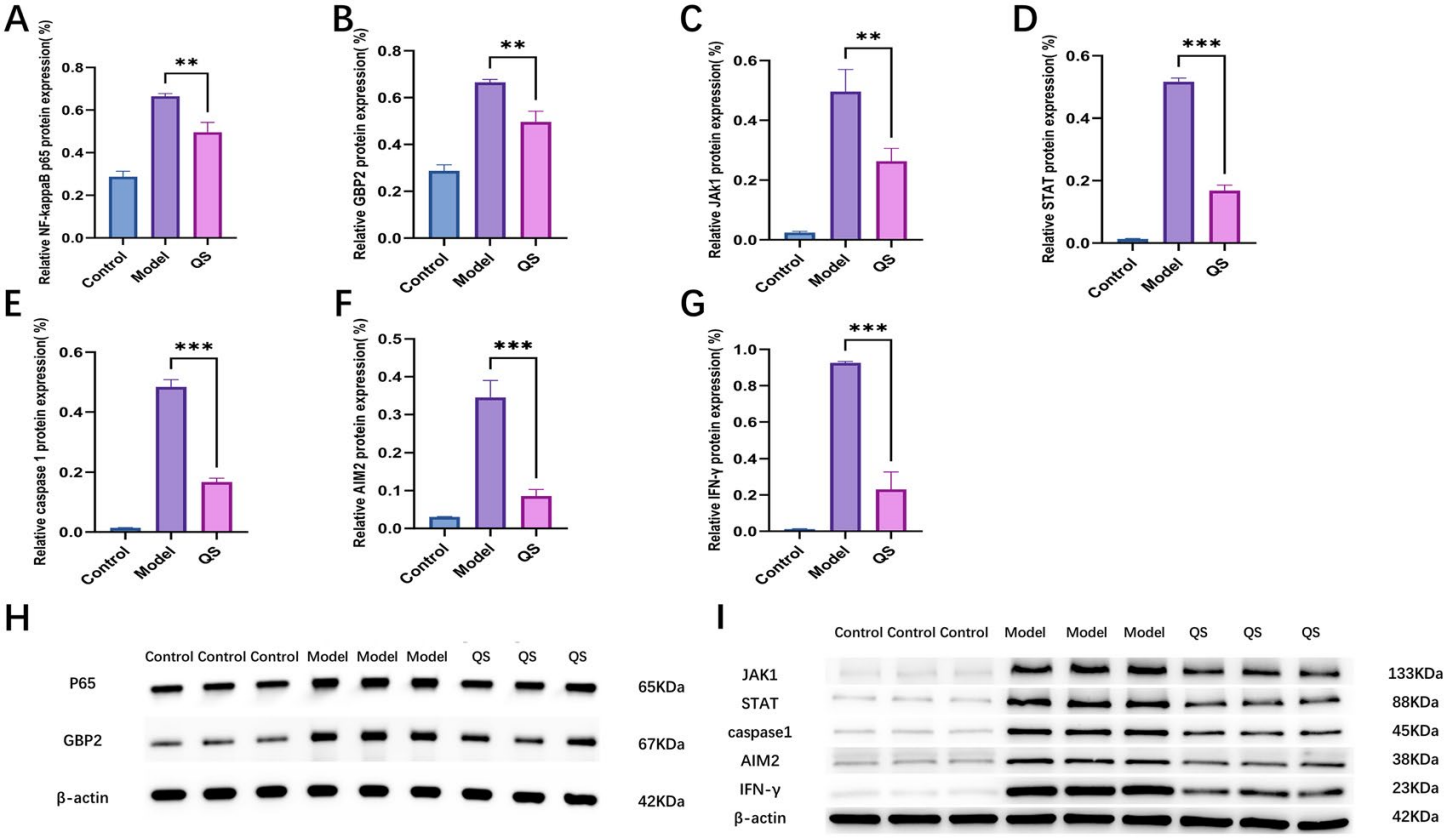
Effects of QS on the expression of renal-related proteins in LN mice. (A–G) The expression of NF-kappaB p65, GBP2, JAK1, STAT, caspase 1, AIM2, and IFN-γ proteins was analyzed through images. (H, I) The expression levels of NF-kappaB p65, GBP2, JAK1, STAT, caspase 1, AIM2, and IFN-γ proteins in the kidneys were detected by Western blot. Statistical significance (*n* = 6) among groups is indicated by asterisks: **p < 0.01; ***p < 0.001.

Compared to the control group, the model group demonstrated significantly higher ratios of target band to reference band grayscale values for NF-kappaB p65, GBP2, JAK1, STAT1, caspase1, AIM2, and IFN-γ-related proteins. In contrast, mice in the QS group exhibited significantly lower ratios for these proteins compared to the model group. The mean differences between the model and QS groups were as follows: NF-kappaB p65: mean difference = −0.164, 95% CI [−0.219, −0.109], Cohen’s *d* = 1.25; GBP2: mean difference = −0.145, 95% CI [−0.200, −0.090], Cohen’s *d* = 1.09; JAK1: mean difference = −0.169, 95% CI [−0.203, −0.135], Cohen’s *d* = 1.25; STAT1: mean difference = −0.349, 95% CI [−0.377, −0.322], Cohen’s *d* = 9.89; caspase1: mean difference = −0.317, 95% CI [−0.355, −0.279], Cohen’s *d* = 8.83; AIM2: mean difference = −0.260, 95% CI [−0.307, −0.213], Cohen’s *d* = 6.20; IFN-γ: mean difference = −0.694, 95% CI [−0.782, −0.607], Cohen’s *d* = 8.13 (Figure 7(A–I)). These results suggest that QS may alleviate LN symptoms by suppressing GBP2 expression and inhibiting the NF-kappaB pathway.

### 3.9. Molecular docking and MM-GBSA binding energy calculation

**Figure 8.**
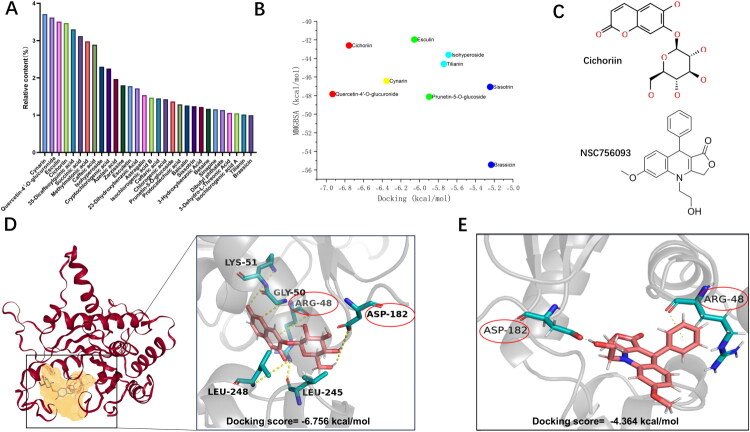
Molecular docking of main components of Qingre Kasen granules and NSC756093 with key proteins, and MMGBSA binding energy calculation. (A) Relative content of main components in QS. (B) Scatter plot of molecular docking and MMGBSA binding energy between main components of QS and GBP2. (C) Molecular structure diagrams of Cichoriin and the GBP1 inhibitor NSC756093. (D) Predicted GBP2 protein binding pocket and docking conformation of cichoriin with GBP2 protein. (E) Docking conformation of NSC756093 with GBP2 protein.

The main components were identified based on preliminary qualitative analysis of QS from our research group (Figure 8(A)). Molecular docking and MMGBSA binding energy calculations between GBP2 protein and the components of QS, as well as the inhibitor NSC756093, were performed using Schrödinger software. Components with a docking score lower than −5.000 kcal/mol and an MMGBSA binding energy lower than −40 kcal/mol were selected, resulting in nine compounds (cynarin, quercetin-4′-O-glucuronide, esculin, cichoriin, isohyperoside, prunetin-5-O-glucoside, sissotrin, tilianin, and brassicin) (Figure 8(B)). By comparing the binding sites of these components with GBP2, we observed that the binding pose of cichoriin with GBP2 exhibits a high degree of overlap with that of NSC756093. Cichoriin formed intermolecular hydrogen bonds with amino acid residues LYS51, GLY50, ASP182, LEU284, and LEU245 of GBP2, and established π–π interactions with ARG48, yielding a docking score of −6.756 kcal/mol (Figure 8(C)). NSC756093 forms intermolecular hydrogen bonds with the amino acid residue ASP182 of GBP2 and establishes π–π interactions with ARG48, the docking score is −4.364 kcal/mol (Figure 8(D)).

### 3.10. Molecular dynamics simulation

**Figure 9.**
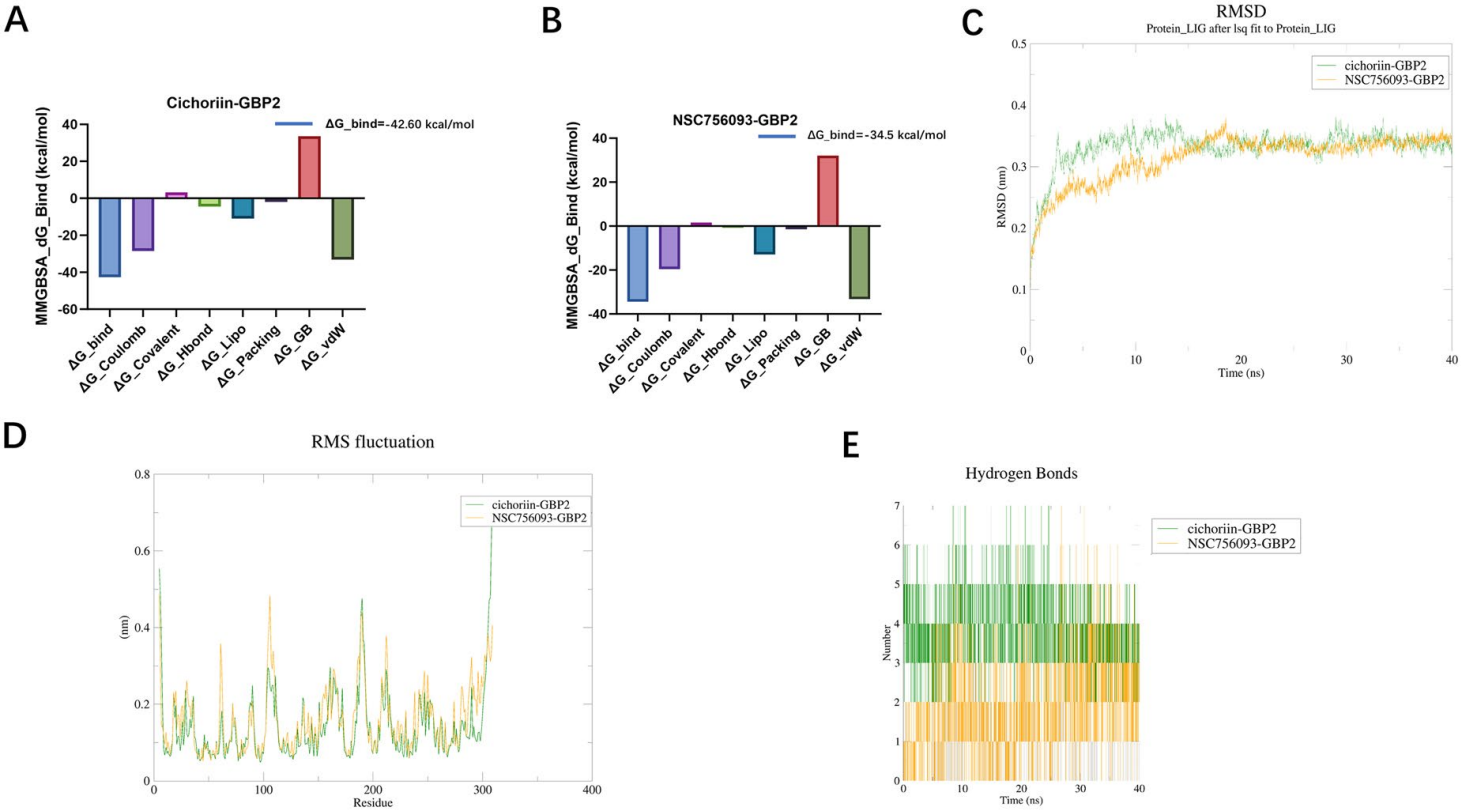
(A) Bar graph of binding free energy between GBP2 and NSC756093 calculated by MMGBSA. (B) Graph showing the root mean square fluctuation (RMSF) of cichoriin–GBP2 complex and NSC756093–GBP2 complex. (C) Graph showing the change of root mean square deviation (RMSD) of cichoriin–GBP2 complex and NSC756093–GBP2 complex. (D) Schematic diagram of structural hydrogen bonds in cichoriin–GBP2 complex and NSC756093–GBP2 complex. (E) Bar graph of binding free energy between GBP2 and cichoriin calculated by MMGBSA.

In MMGBSA calculations, a binding energy greater than −30 kcal/mol indicates weak binding, −30 to −50 kcal/mol indicates moderate binding, and less than −50 kcal/mol signifies strong binding. The binding free energy between cichoriin and GBP2 was −42.60 kcal/mol (Figure 9(A)), while that of NSC756093 with GBP2 was −34.5 kcal/mol (Figure 9(B)), both falling within the moderate binding range. However, cichoriin exhibited stronger binding.

Molecular docking was performed on the cichoriin–GBP2 complex and the NSC756093–GBP2 complex using Gromacs software. The results showed that regarding the RMSF (root mean square fluctuation) value, a smaller change in the value indicates better binding stability of the complex. The cichoriin–GBP2 complex exhibited lower RMSF fluctuations compared to the NSC756093–GBP2 complex, indicating enhanced local structural stability (Figure 9(C)). For the RMSD (root mean square deviation) value, a smaller change also indicates better binding stability of the complex. The RMSD of the cichoriin–GBP2 complex fluctuated slightly between 0.3 and 0.35 nm, and the fluctuation amplitude was slightly smaller than that of NSC756093–GBP2 (Figure 9(D)), which indicates that the binding of cichoriin to GBP2 protein is more stable. In the hydrogen bond diagram, a higher value indicates more hydrogen bonds formed by the system at the corresponding time point, and more hydrogen bonds mean a better binding effect. The cichoriin–GBP2 complex had a higher value and more binding hydrogen bonds, making it easier to form a lasting and specific binding complex (Figure 9(E)).

### 3.11. ADMET analysis of cichoriin molecule

**Figure 10.**
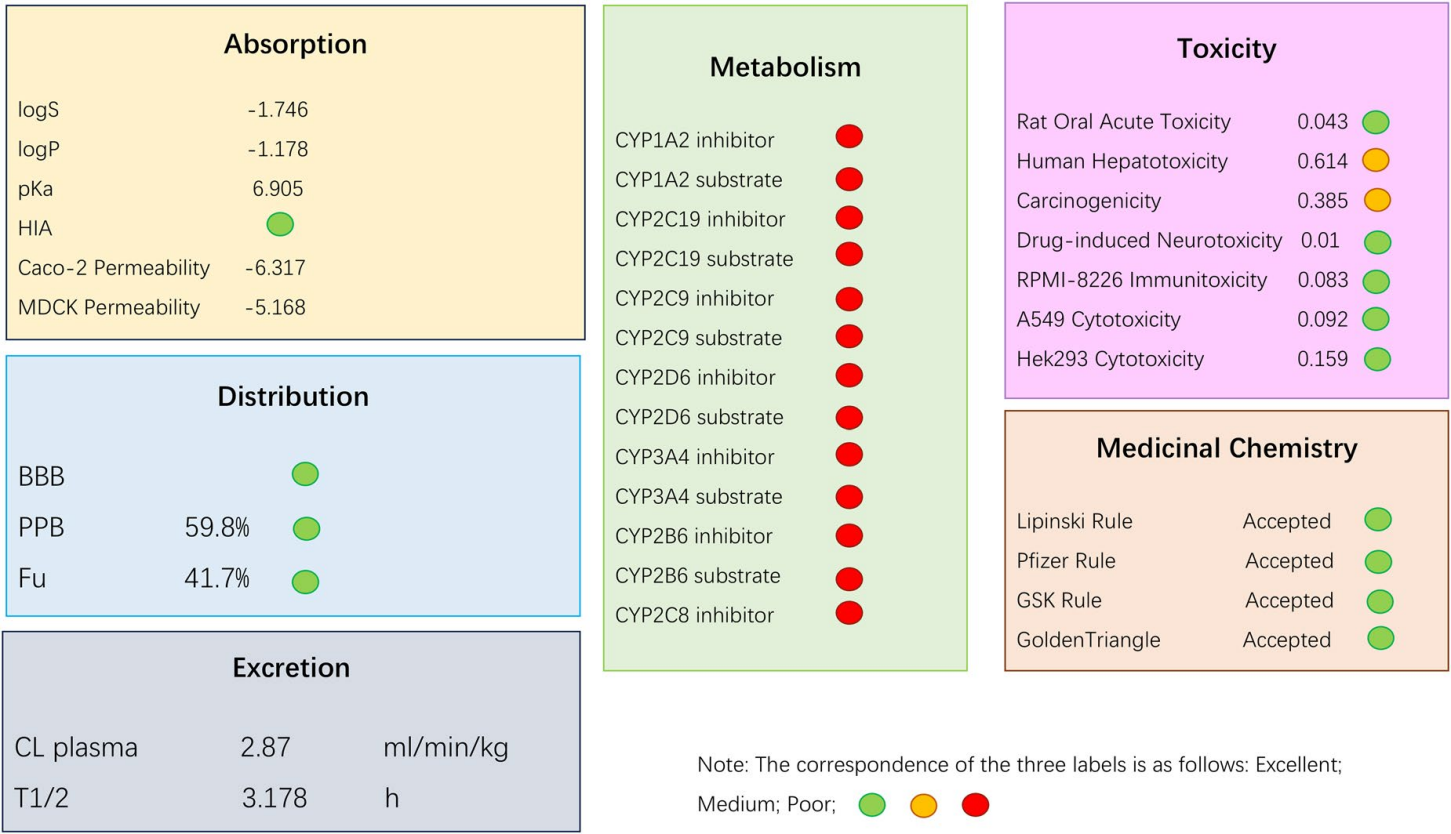
ADMET analysis of cichoriin.

The ADMET prediction results of cichoriin showed that it has moderate water solubility (log*S* = −1.746) and strong hydrophilicity (log*P* = −1.178). The prediction of human intestinal absorption (HIA) was good. The permeability of Caco-2 cells was moderate (log*P*app = −6.317), while the permeability of MDCK cells was high (log*P*app = −5.168). It has good blood–brain barrier (BBB) penetration, suggesting that it may have potential central nervous system activity. The plasma protein binding (PPB) rate was 59.8% and the free drug fraction (Fu) was 41.7%, indicating that it has relatively optimal distribution characteristics, which is beneficial for drug molecules to reach the target. Cichoriin is not an inhibitor or substrate of major cytochrome P450 (CYP450) enzymes such as CYP1A2 and CYP2C19, indicating that it has no significant risk of drug–drug interactions (DDIs). The plasma clearance rate was 2.87 mL/min/kg, and the half-life was 3.178 h, with a moderate elimination rate, so a reasonable administration scheme needs to be designed. The acute oral toxicity, neurotoxicity, immunotoxicity, and cytotoxicity in rats were all low (marked in green), showing good safety; however, human hepatotoxicity (0.614) and carcinogenicity (0.385) were at moderate levels (marked in yellow), requiring further evaluation of long-term risks. Cichoriin conforms to the Lipinski, Pfizer, GSK, and Golden Triangle druggability rules (marked in green) and complies with the principles of druggability (Figure 10).

In summary, cichoriin exhibits favorable ADMET properties and is predicted to have good development potential.

**Figure 11.**
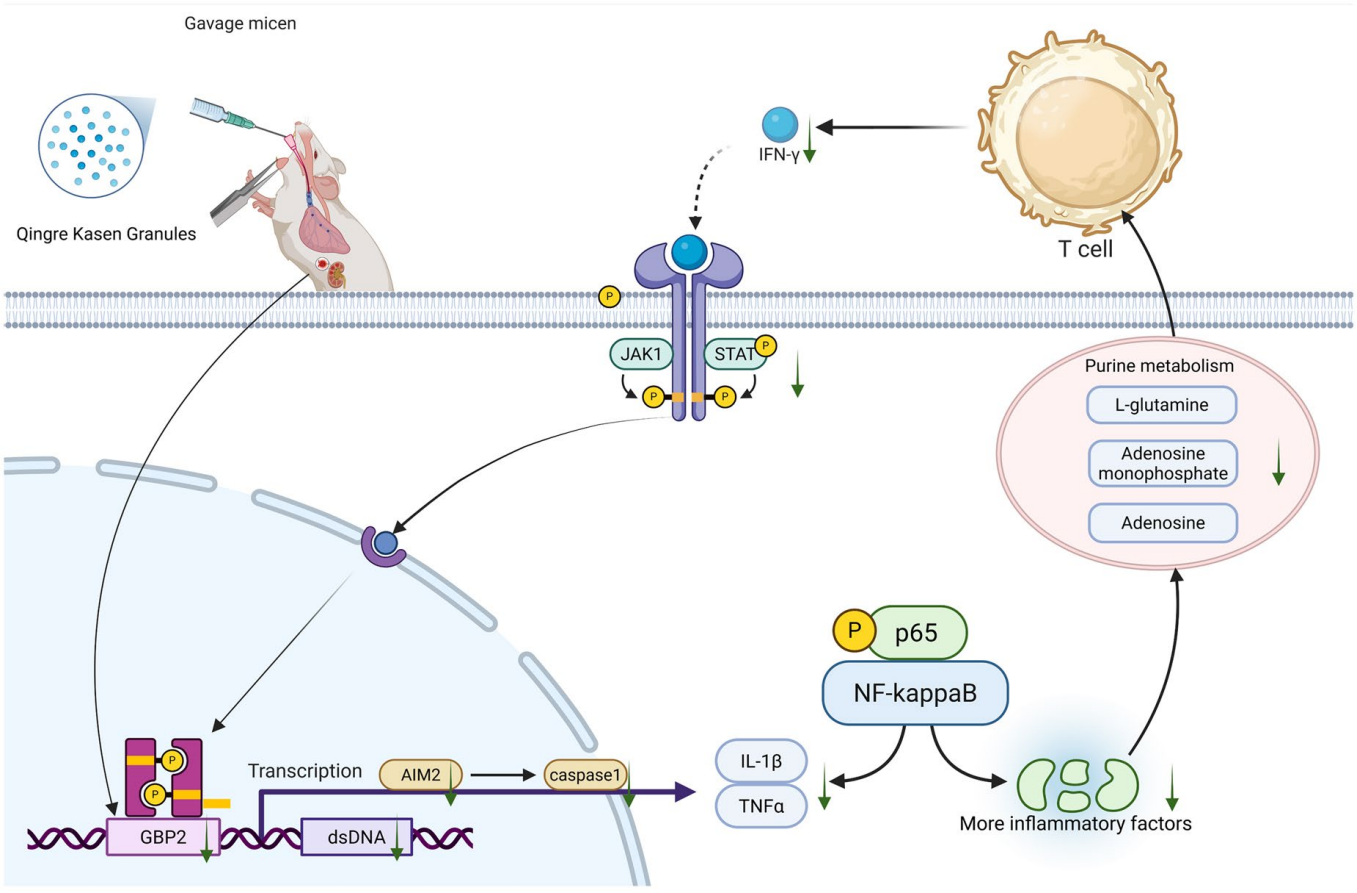
The mechanism of action of Qingre Kasen granules in treating LN mice.

## 4. Discussion

LN, one of the most severe and prevalent complications of SLE, primarily affects the kidneys. This can significantly impair renal function and, in severe cases, may even progress to renal failure [^21^]. The patient’s body produces large numbers of autoantibodies, which form immune complexes that deposit in the kidneys, leading to a series of inflammatory responses [^22^]. Currently, the main clinical treatment for LN is the combined use of glucocorticoids and immunosuppressants. Although this approach can control the disease condition to a certain extent, it is accompanied by numerous adverse reactions, such as infections, osteoporosis, and a relatively high recurrence rate. Therefore, there is an urgent need to explore safer and more effective treatment regimens [^23^]. As the main component of QS, chicory has been shown in previous studies to attenuate the expression of the NF-kappaB signaling pathway and treating inflammatory diseases [^24^,^25^]. In this study, through verification by external datasets and establishment of LN mouse models, GBP2 was identified as a potential therapeutic target for LN, with its mechanism of action related to the NF-kappaB signaling pathway. Meanwhile, it was confirmed that QS had a positive therapeutic effect on MRL/Lpr mice. Metabolomic analysis suggested that the therapeutic effect of QS on LN might be associated with the purine metabolism pathway. Adenosine, AMP, and glutamine were identified as key metabolites in this process, suggesting their important roles in the mechanism of QS in treating LN. Finally, through molecular docking, MD simulation, and MMGBSA binding energy calculation, cichoriin was predicted to be a potential active component of QS for the treatment of LN. Additionally, ADMETLab 3.0 predicted that cichoriin possesses favorable properties.

QS has the effect of alleviating the symptoms of LN mice. Anti-dsDNA antibody is a specific marker for SLE and LN, while ANA serves as an initial screening indicator for autoimmune diseases, particularly SLE [^26^]. The increase in the levels of dsDNA and ANA in an organism indicates a more severe autoimmune reaction [^27^]. In this study, after administering QS to LN mice, the levels of anti-dsDNA antibody, ANA antibody, IL-1β, and TNF-α in their bodies were significantly reduced. Meanwhile, in the pathological sections, we observed that the QS group exhibited significantly less renal damage, reduced fibrosis, and a lower number of inflammatory cells compared to the model group. Additionally, the expression of the key protein GBP2 in renal tissues was markedly downregulated. These data suggest that QS can effectively relieve the symptoms of LN.

QS may exert an effect on LN by inhibiting the GBP2/NF-kappaB signaling pathway. GBP2 is a member of the guanylate-binding protein (GBP) family and is a type of protein induced by IFN-γ [^28^]. GBP2 has functions such as antiviral and antibacterial activities, regulation of apoptosis, and participation in intracellular signal transduction pathways [^29^]. In the kidneys of LN patients, besides glomerular and tubulointerstitial damage, various inflammatory cytokines are produced, accompanied by abnormal activation of inflammation-related pathways such as NF-kappaB [^30^]. Based on existing studies, the overexpression of GBP2 can promote the activation of AIM2 during bacterial infection [^31^]. The AIM2 inflammasome detects dsDNA in the cytosol and induces caspase-1-dependent pyroptosis as well as the release of inflammatory cytokines IL-1β and IL-18, thereby activating the NF-kappaB signaling pathway [^32^]. The activated NF-kappaB signaling pathway, in turn, drives the transcription of other pro-inflammatory mediators such as TNF-α and IL-6, forming a positive feedback loop that exacerbates local inflammatory damage in the kidneys [^33^]. These inflammatory cytokines also feedback on back on CD4^+^ T cells, promoting their differentiation and forming a cycle [^34^]. In the experiment, the expression levels of genes such as IFN-γ, GBP2, AIM2, and Caspase1 in the QS group were lower than those in the model group, and the phosphorylation level of NF-kappaB p65 decreased simultaneously, indicating that the drug may inhibit the amplification of inflammatory signals by targeting the GBP2–NF-kappaB axis. Thus, we speculate that QS may affect the symptoms of LN mice by inhibiting the GBP2/NF-kappaB signaling pathway.

QS alleviates the condition by inhibiting the release of inflammatory cytokines, suppressing purine metabolism, and consequently reducing the activation of CD4^+^ T cells as well as the overexpression of GBP2. In the metabolomic analysis, we observed aberrant signaling in the purine metabolism pathway. Purine metabolism is closely associated with inflammatory cytokines, and excessive inflammatory cytokines can further disrupt purine metabolism homeostasis by promoting renal uric acid production [^35^,^36^]. Purine metabolism provides nucleotide precursors such as adenosine triphosphate (ATP) and guanosine triphosphate (GTP), directly influencing the division and function of CD4^+^ T cells [^37^,^38^]. In this process, glutamine undergoes deamination to release ammonia, serving as a nitrogen donor for nucleotide synthesis [^39^]. ATP hydrolysis to AMP provides essential energy for cellular activities, and AMP is further dephosphorylated to adenosine, which continues to participate in various metabolic processes [^40^,^41^]. In immune cells, CD4^+^ T cells play a crucial role in the pathogenesis of SLE and are critical for maintaining immune homeostasis [^42^,^43^]. CD4^+^ T cells play a critical role in defending against infections, and a lower level of CD4^+^ T lymphocytes indicates a defect in the host immune system [^44^]. Based on the immune cell infiltration analysis of LN patients and healthy controls, we found that the infiltration proportions of naïve CD4^+^ T cells (downregulation) and resting memory CD4^+^ T cells (upregulation) in the disease group changed significantly. When pathogens invade the body, antigen-presenting cells transmit pathogen signals to CD4^+^ T cells [^45^]. The activated CD4^+^ cells release IFN-γ and transform into effector T cells or memory T cells [^46^]. IFN-γ binds to the interferon-γ receptor (IFNγR) on the surface of macrophages [^47^], activates the downstream JAK1/STAT signaling pathway, and induces the transcriptional expression of interferon-stimulated genes (ISGs) [^48^]. Among them, GBP2 as a key member of ISGs, the expression level of GBP2 is significantly upregulated under the stimulation of IFN-γ [^49^]. Thus, a vicious cycle is formed. In the Western blot analysis of kidneys from LN mice, the upregulation of genes including IFN-γ, JAK1, STAT, and GBP2 in the model group further confirmed this finding. However, with QS intervention, the expression of these genes was downregulated, indicating that QS disrupts this vicious cycle and alleviates the symptoms of LN.

Thus, identifying targeted components for GBP2 is critical. Based on the molecular docking and MMGBSA results of the main components of QS with the GBP2 protein, nine components with favorable scores were identified. Subsequent comparison with the binding site of the effective inhibitor NSC756093–GBP2 revealed that cichoriin is considered a potential therapeutic agent, exhibiting a high docking score of −6.756 kcal/mol. In contrast, the docking score of NSC756093 with GBP2 was slightly lower, at −4.364 kcal/mol [^50^]. Molecular docking showed that the binding sites of cichoriin to GBP2 include ASP182 and ARG48, which highly overlap with the binding sites of the known inhibitor NSC756093 in the homologous region of GBP2. Kinetic simulations showed that the cichoriin–GBP2 complex exhibited lower RMSD and higher hydrogen bond occupancy compared to the NSC756093–GBP2 complex, indicating stronger stability. Binding free energy calculations based on MM/GBSA further supported these findings. We therefore hypothesize that cichoriin ameliorates LN symptoms by regulating GBP2 and influencing the NF-kappaB signaling pathway. As a bioactive coumarin, cichoriin has been reported to improve hepatorenal metabolic dysfunction and pathological features, exhibit antiviral effects, and exert anti-inflammatory and antioxidant activities through the NF-kappaB and AMPK pathways [^51^,^52^]. This echoes with the regulatory mechanism of the GBP2/NF-kappaB signaling axis identified in this study.

*In silico* ADMET predictions revealed that cichoriin exhibits significant drug development potential based on its ADMET properties. Its balanced hydrophilic–lipophilic profile, low PPB rate (59.8%), and non-CYP450-mediated metabolism collectively ensure high bioavailability and low risk of clinical DDIs. Notably, the differential permeability observed between the Caco-2 and MDCK models (log*P*app −6.317 vs. −5.168) suggests that intestinal absorption efficiency may limit oral bioavailability, necessitating prodrug design to overcome this bottleneck. Despite favorable toxicity profiles in rats, the moderate risks of human hepatotoxicity and carcinogenicity (probability values > 0.3) still require mechanistic investigations and long-term *in vivo* genotoxicity assays.

Compared with current standard treatment drugs, QS reduced serum anti-dsDNA levels by 26.55 pg/mL (Cohen’s *d* = 1.95) and alleviated renal interstitial fibrosis. Its therapeutic effect was comparable to that of MMF in the same model (anti-dsDNA reduction: ∼25 pg/mL, Cohen’s *d* = 2.10) [^53^]. Notably, QS demonstrated favorable safety profiles in our preclinical model: it showed low acute toxicity and no obvious adverse reactions – an advantage over MMF, which carries risks of gastrointestinal toxicity and bone marrow suppression [^54^]. Although direct comparative clinical trials are still needed, these findings suggest that QS may offer a complementary or alternative therapeutic strategy with potential safety advantages for LN management.

In conclusion, QS may regulate the purine metabolism pathway to affect the activation and differentiation of CD4^+^ T cells; simultaneously, they inhibit the expression of proteins related to the GBP2–NF-kappaB signaling pathway, reduce the production of pro-inflammatory cytokines, and thereby modulate the abnormal circulatory state of CD4^+^ T cells to ameliorate symptoms of LN. As a key component, cichoriin has favorable ADMET properties and is a candidate molecule with great development value, but it may require targeted optimization of absorption and systematic evaluation of long-term safety.

Despite the encouraging findings of this study, the following limitations should be noted. First as a core pathway regulating the body’s inflammatory response, immune homeostasis, and cell survival, NF-kappaB signaling pathway plays a significant role in various normal physiological processes (such as immune cell development and tissue repair). If it is subject to extensive and nonspecific inhibition, off-target effects may occur – for instance, interfering with normal immune defense functions and increasing the risk of infection, or affecting intestinal barrier homeostasis and hematopoietic function [^55^,^56^]. However, in this study, the inhibition of NF-kappaB by QS may not be a broad-spectrum intervention. On one hand, cichoriin exerts its effects by binding to specific sites (ASP182, ARG48) of GBP2. Its binding sites highly overlap with those of the known inhibitor NSC756093 in the homologous region of GBP2, and MD simulations show that its complex with GBP2 has strong stability. This suggests that it may indirectly regulate the NF-kappaB pathway by specifically targeting GBP2, rather than directly inhibiting the core molecules of NF-kappaB. On the other hand, in the experiment, the QS group only significantly downregulated the inflammatory factors related to LN pathology (such as IL-1β, TNF-α, etc.), and no obvious acute toxicity or immune function abnormalities were observed. This also indicates from the side that it may precisely intervene in the pathologically activated part of the ‘GBP2–NF-kappaB’ axis, thereby exerting the therapeutic effect while reducing the interference with the normal physiological functions of NF-kappaB. Nevertheless, this speculation still needs further experimental verification. For example, by detecting the impact of cichoriin on the basic activity of the NF-kappaB pathway in normal cells, or evaluating its effect on other physiological processes regulated by NF-kappaB (such as mucosal immunity), to clarify the specificity of its inhibition on NF-kappaB and the potential off-target risks.

Second, the sample size in animal experiments (*n* = 6 per group) is relatively small, which is lower than that determined by the preset power analysis, although statistically significant results were obtained for key endpoint indicators (such as serum biomarkers and histopathology). Additionally, despite employing cross-validation, external dataset validation, and multiple algorithms (LASSO, RandomForest) to enhance reliability, false positive findings cannot be completely ruled out in high-dimensional data. Furthermore, as this study is based on preclinical experiments, the mechanisms of action of the predicted target (GBP2) and candidate drug (cichoriin) require further verification and refinement. For the clinical translation of cichoriin, *in vivo* evaluations in higher animal models (such as non-human primates) are necessary to determine its safety margin, especially considering the long-term treatment requirements for LN.

Our next steps will involve expanding the sample size and further validating the mechanism of action between GBP2 and cichoriin through *in vivo* and *in vitro* experiments. We will advance from murine models to human biopsy validation – by detecting the expression of GBP2 in renal biopsy tissues of LN patients with different pathological types and clarifying its correlation with disease activity. Additionally, we will optimize the machine learning models used in the study, expand the scale of external validation datasets, and enhance the reproducibility and clinical applicability of AI/ML models in identifying LN biomarkers and predicting treatment responses. And explore the potential of combined collaboration between QS and currently major therapeutic drugs (e.g., MMF, glucocorticoids, etc.).

## 5. Conclusions

In this study, by integrating machine learning and bioinformatics, we systematically screened out five characteristic DEGs significantly associated with LN. Through validation using external datasets, GBP2 was retained. In the analysis of immune cell infiltration in LN patients, we noticed that the pathogenesis of LN is related to CD4^+^ T cells. Subsequently, by establishing an LN mouse model, we confirmed that QS can significantly ameliorate renal pathological damage in LN, and its therapeutic mechanism may be related to affecting purine metabolism and regulating the GBP2/NF-kappaB axis (Figure 11). Finally, through chemoinformatic calculations, we predicted that cichoriin may be a potential active component for LN treatment and has certain potential development value. Certainly, this study still has some limitations, including a small sample size, the possibility of false positives in the data, and the need for more rigorous verification and investigation of certain mechanisms. We will conduct in-depth research in future studies to provide more robust evidence and address such issues. This study established a ‘biomarker–animal model–active component’ trinity research system, which not only verified the efficacy of the traditional formula, but also pioneered a modern research and development pathway for the repurposing and discovery of natural product drugs. Through the integration of computational biology, machine learning, and experimental validation, it not only provides a novel molecular target (GBP2) for the precise diagnosis and treatment of LN, but also successfully transforms QS from an empirical traditional Chinese medicine into a source of novel immunomodulators with validated targets, and reveals the development value of cichoriin as a natural small-molecule drug.

## Supplementary Material

Supplementary Table S1 and S2.docx

## Data Availability

The custom R scripts used for bioinformatics analyses in this study are only partially available due to institutional policy restrictions. Specifically, the code for immune infiltration analysis has been deposited in the GitHub repository (link: https://github.com/KaojiangZhu/Immune-infiltration-analysis). However, the ‘Methods’ section describes the complete analysis workflow in sufficient detail to enable result reproduction. For specific inquiries regarding the implementation, please contact the corresponding author directly.
