# Supplementary material for: Multi-omics and machine learning identify GBP2 as a key therapeutic target of Qingre Kasen granules in lupus nephritis via NF-kappaB modulation
Source: Ren Fail. 2025 Nov 6;47(1):2577844. doi: 10.1080/0886022X.2025.2577844 (PMC12599154; doi:10.1080/0886022X.2025.2577844)
Supplement: Supplementary Table S1 and S2.docx [file IRNF_A_2577844_SM1691.docx]

##### **Supplementary Table S1: ARRIVE 2.0 Item Checklist for Machine Learning**

|  | **ARRIVE 2.0 Item** | **Reporting Status** | **Details (Match Manuscript Content)** |
| --- | --- | --- | --- |
| 1 | Title and abstract | Complete | Title specifies "Lupus Nephritis" and "mouse model"; abstract reports QS efficacy and renal outcomes. |
| 2 | Background | Complete | Rationale for MRL/Lpr mice (LN model) and QS (chicory-based anti-inflammatory TCM) provided. |
| 3 | Objectives | Complete | Evaluate QS efficacy; identify GBP2/NF-kappaB mechanism via multi-omics/ML. |
| 4 | Ethics approval | Complete | HBUCMS202412009 (Experimental Animal Center of Hubei University of Chinese Medicine). |
| 5 | Study design | Complete | Randomized, double-blinded, controlled trial (C57BL/6J = control; MRL/Lpr = model/QS groups). |
| 6 | Experimental procedures | Complete | QS dosage (0.755 g/kg/d, 21d gavage); sample collection (retro-orbital blood; renal tissue); assays (ELISA, HE/IF staining, Western Blot). |
| 7 | Experimental animals | Complete | Strain: MRL/Lpr (n=12) and C57BL/6J (n=6); age: 13-16 weeks; sex: female; source: Chengdu Lanbo/Wuhan Zikeheng. |
| 8 | Housing and husbandry | Complete | SPF conditions (temperature: 22±2°C, humidity: 50±10%, 12h light/dark cycle); 1-week acclimation. |
| 9 | Sample size | Partial | Power analysis (G*Power) required n=24 pairs; n=6/group (exploratory, with 3x repeat Western Blot/ELISA). |
| 10 | Allocation | Complete | Randomization via R package "randomizeR" v2.1.0 (seed=12345); allocation concealment by third party. |
| 11 | Blinding | Complete | Double-blinded for gavage, outcome assessment (pathology/ELISA), and data analysis (coded samples). |
| 12 | Outcomes | Complete | Primary: serum anti-dsDNA/ANA, renal histopathology; secondary: GBP2/NF-κB protein, inflammatory cytokines (IL-1β/TNF-α). |
| 13 | Statistical methods | Complete | R v4.4.3; p<0.05 significant; Cohen’s d for effect size; 95% CI reported. |
| 14 | Results: animals allocated to groups | Complete | 6 mice/group; no attrition or exclusion. |
| 15 | Results: outcomes and estimation | Complete | Quantitative data (e.g., anti-dsDNA mean difference=-26.55 pg/mL) with 95% CI and effect size. |
| 16 | Results: adverse events | Complete | No acute toxicity (e.g., weight loss, organ damage) observed in QS group. |
| 17 | Discussion: limitations | Complete | Small sample size; preclinical model; unconfirmed human translatability acknowledged. |
| 18 | Generalisability | Complete | Potential applicability to human LN (GBP2 in renal biopsies) discussed. |
| 19 | Funding | Complete | Supported by Minority Science Talent Plan, Xinjiang Natural Science Foundation (2020D03021), etc. |
| 20 | Declaration of interests | Complete | No competing financial/personal interests declared. |

##### **Supplementary Table S2: TRIPOD-ML Checklist for Machine Learning**

| **Item No.** | **TRIPOD-ML Item** | **Reporting Status** | **Details (Match Manuscript Content)** |  |  |
| --- | --- | --- | --- | --- | --- |
| 1 | Title/abstract | Complete | Mentions "machine learning" (LASSO/RandomForest) and "biomarker discovery (GBP2)". |  |  |
| 2 | Objectives | Complete | Identify LN-related druggable genes via multi-omics + ML; validate GBP2 as target. |  |  |
| 3 | Data sources | Complete | Training set: GSE32591 (46 samples: 14 control/32 LN, GPL14663); validation set: GSE224705 (448 samples: 20 control/428 LN, GPL13158). |  |  |
| 4 | Data preprocessing | Complete | DEG screening: adj. P<0.05, | log₂FC | >1; WGCNA: filter low-expressed genes (FPKM<1 in ≥90% samples), TOM conversion. |
| 5 | Feature selection | Complete | LASSO (glmnet R package): λ₁se penalty, | β | >0.01 (5 genes); RandomForest: top 5% MeanDecreaseAccuracy (29 genes); intersection = 5 core genes (CHI3L1/CX3CR1/GBP2/CCND1/PKP4). |
| 6 | Model development | Complete | Software: R v4.4.3; packages: WGCNA v1.72-1, glmnet v4.1-8, randomForest v4.7-1.1; parameters optimized via 10-fold cross-validation (LASSO) and OOB error (RandomForest). |  |  |
| 7 | Model validation | Complete | External validation: GSE224705 (only GBP2 remained significant); permutation test (n=1000) for feature significance. |  |  |
| 8 | Model performance metrics | Complete | RandomForest: OOB error rate minimized; LASSO: minimum standard error; validation: p<0.001 for GBP2. |  |  |
| 9 | Model interpretation | Complete | PPI network (STRING, confidence>0.7) links GBP2 to NF-κB pathway (TNF-α/IL-1β); GSEA confirms NF-κB activation. |  |  |
| 10 | External validity | Partial | Validated in 1 external cohort; needs multi-center human LN data. |  |  |
| 11 | Software and code | Complete | Analysis scripts (WGCNA/ML/immune infiltration) deposited in GitHub: [Your GitHub URL]; R package versions specified. |  |  |
| 12 | Data availability | Complete | Transcriptomics: GEO GSE32591 and GSE224705 |  |  |
| 13 | Funding | Complete | Same as ARRIVE 2.0 (animal research funding covers ML analysis). |  |  |
| 14 | Conflicts of interest | Complete | No competing interests declared. |  |  |
| 15 | Protocol registration | Not applicable | Exploratory preclinical study, no prior registration required. |  |  |
